# Supplementary material for: Ferroptosis Among the Antiproliferative Pathways Activated by a Lipophilic Ruthenium(III) Complex as a Candidate Drug for Triple-Negative Breast Cancer
Source: Pharmaceutics. 2025 Jul 16;17(7):918. doi: 10.3390/pharmaceutics17070918 (PMC12300231; doi:10.3390/pharmaceutics17070918)
Supplement: Supplementary file 1 [file pharmaceutics-17-00918-s001.zip › pharmaceutics-3737239-supplementary.pdf]

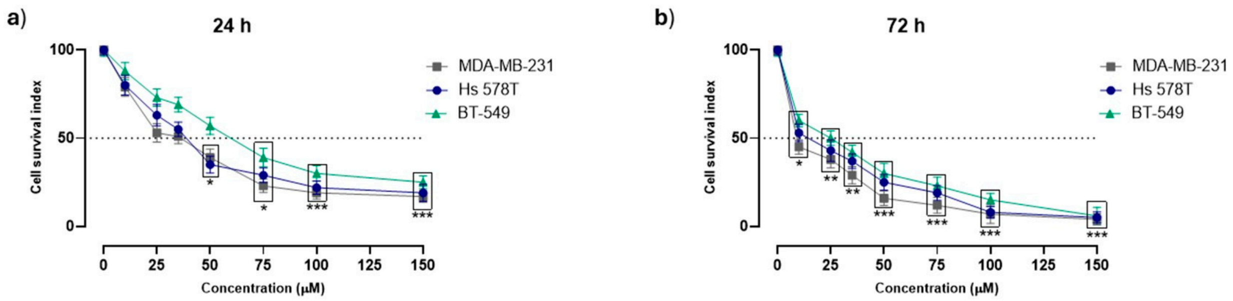

Figure S1. Antiproliferative effects of PalmiPyRu in TNBC cells. Cell survival index, evaluated by the MTT assay and live/dead cell ratio analysis, for TNBC cell lines (MDA-MB-231, Hs 578T, BT-549), as indicated in legends, after a 24 (**a**) and 72 h (**b**) treatment with different concentrations of PalmiPyRu (range 5→150 μM). Data are plotted in line graphs as percentage of untreated control cells and are reported as mean of three independent experiments ± SEM (n = 15). $^*p < 0.05$ with respect to control cells; $^{**}p < 0.01$ with respect to control cells; $^{***}p < 0.001$ with respect to control cells.
